# How do mammals convert dynamic odor information into neural maps for landscape navigation?

**DOI:** 10.1371/journal.pbio.3002908

**Published:** 2024-11-21

**Authors:** Anantu Sunil, Olivia Pedroncini, Andreas T. Schaefer, Tobias Ackels

**Affiliations:** 1 Sensory Dynamics and Behaviour Lab, Institute of Experimental Epileptology and Cognition Research, University of Bonn Medical Center, Bonn, Germany; 2 Sensory Circuits and Neurotechnology Laboratory, Francis Crick Institute, London, United Kingdom; 3 Department of Neuroscience, Physiology and Pharmacology, University College London, London, United Kingdom

## Abstract

Odors are transported by seemingly chaotic plumes, whose spatiotemporal structure contains rich information about space, with olfaction serving as a gateway for obtaining and processing this spatial information. Beyond tracking odors, olfaction provides localization and chemical communication cues for detecting conspecifics and predators, and linking external environments to internal cognitive maps. In this Essay, we discuss recent physiological, behavioral, and methodological advancements in mammalian olfactory research to present our current understanding of how olfaction can be used to navigate the environment. We also examine potential neural mechanisms that might convert dynamic olfactory inputs into environmental maps along this axis. Finally, we consider technological applications of odor dynamics for developing bio-inspired sensor technologies, robotics, and computational models. By shedding light on the principles underlying the processing of odor dynamics, olfactory research will pave the way for innovative solutions that bridge the gap between biology and technology, enriching our understanding of the natural world.

## Abstract 2

Odors contain complex spatiotemporal information that guide animal behavior, but how this information is processed is not fully understood. This Essay discusses recent advances in mammalian olfactory research to present our current understanding of how olfaction can be used to navigate the environment.

## Introduction

In natural settings, odors typically occur as complex mixtures of often hundreds of different chemical compounds rather than isolated entities, comprising varied compositions and concentrations of volatile components [1]. These mixtures are transported through complex, often turbulent, airflow, forming odor plumes that, in addition to their chemical complexity, are characterized by a rich temporal structure [2–5]. It has been proposed that the spatial and temporal patterns within odor plumes contain valuable information regarding the location, distance, and composition of odor sources [6–9]. Recent studies indicate that, against the traditional dogma of olfaction only acting as a slow sense [10–15], the mammalian olfactory system possesses a high bandwidth capacity, crucial for detecting and interpreting rapid changes in odor concentration over time inherent to natural odor plumes [16–21]. It is becoming increasingly evident that the olfactory system processes these odor landscapes to interpret spatial orientation information, providing animals with contextual cues about their position within a broader environmental framework. Although evidence points towards the hippocampal formation as a major processing center of this information, the detailed mechanisms and neural correlates of this spatiotemporal mapping remain incompletely understood, posing major questions in the field.

In this Essay, we discuss the high temporal bandwidth capacity of the mammalian olfactory system and delve into the neural correlates that could facilitate translating olfactory inputs into environmental maps. We also highlight some technological applications of sensing and processing dynamic odors, underscoring the need for a better understanding of the processing of odor dynamics.

## Gathering spatial information through olfaction

Animals trained to locate an odor source implement various strategies to do so, and adaptively switch between them depending on the task, environment, level of learning, and other behavioral factors. Such adaptations might include adjusting their speed, changing direction more efficiently in response to fluctuating odor signals, or their ability to discriminate between closely spaced odor sources. The continuous exposure to odor dynamics might not only aid animals in refining their navigation efficiency but also enhance their ability to accurately detect and localize the source over time. Fruit flies, for example, have been found to use timing of odor encounters to navigate through complex plumes [22]. Insects are also capable of detecting odor onset-asynchrony with high precision [23]. For mammals, this requires the ability to process dynamic odor signals in a frequency range exceeding that of the respiration cycle, particularly for extending sensory perception over a large range of distances. Studies harnessing the temporal precision of light stimulation have shown with optogenetics that mice can discriminate odors based on timing differences as small as 10 ms within the sniff cycle, and that glomeruli activated earlier in the sequence have a stronger impact on perception [24–26]. These features offer distinct advantages to crepuscular (those active during twilight) and nocturnal animals. High olfactory bandwidth enables these animals to efficiently navigate and exploit their environment, leveraging olfactory cues for locating food, detecting predators, or identifying mates, even under conditions of limited visibility.

This ongoing interaction with varying odor concentrations and patterns allows animals to develop a more nuanced understanding of their environment based on immediate sensory feedback. A common method used by animals involves odor trail tracking, such as following urine scent marks [27,28]. As they move along a trail, animals are thought to utilize the concentration gradient surrounding the trail and perform inter-naris [29] and inter-sniff comparisons [30]. This is augmented by scanning movements across the path, similar to casting movements observed in insects [31–34]. Environmental parameters such as temperature and humidity can also influence the sampling strategies, as recently shown by a study on dogs [35]. Besides following odor trails deposited on the ground, animals (and rodents in particular) may also need to orient themselves relative to distant odor sources, avoid predators, or search for stationary items like buried food, which do not leave a trail [36]. In these cases, they track airborne odor plumes, characterized by high-frequency fluctuations and dynamically changing shapes driven by airflow turbulence, which likely presents a more complex challenge [37,38]. Cues in temporally complex odor plumes are used for navigation by using coarse encoding of odor concentrations across multiple sensors and time points, with nonlinear receptor responses emphasizing rare high-concentration samples, which provide the most information about the source location [39,40]. Detecting and processing concentration gradients and temporal shifts in odor plumes can aid mice and rats to discern their movements relative to the source of the odor, showing that they can effectively map their surroundings through olfactory cues [41,42]. The integration of temporally complex olfactory information as a result might facilitate animals to construct an internal representation of their external world, answering fundamental navigational questions such as “Where am I in relation to key environmental features or other organisms?” This highlights the capacity of olfaction as a sensory modality to acquire spatial information. While natural odor stimuli do include high-frequency components detectable by the peripheral olfactory system, it has not been established to what extent rodents actually rely on these temporal features versus integrating odor cues with self-motion and other environmental landmarks during localization tasks. It has been demonstrated that rodents can rapidly switch from relying solely on olfactory cues to incorporating spatial memory when navigating to odor sources [43]. This flexibility suggests that rodents use a combination of olfactory information and learned spatial cues, depending on the context and availability of information. Meanwhile, studies have shown that humans can also utilize olfactory cues for navigation [44] and detect odor onset latencies with remarkable temporal precision of 120 ms [45,46].

Olfaction is also central to chemical communication between individuals or groups of animals, such as mice, extending its role beyond pinpointing food sources. Continuous active sampling of the olfactory environment through sniffing gathers essential information about the presence of other organisms, including both conspecifics and potential predators [47,48]. By detecting chemical signals released or deposited by other animals, individuals can ascertain various biological and social cues, such as the social or reproductive status of potential mates, the territorial markings of conspecifics, or the warning signals indicative of predators [49]. This steady flow of olfactory information forms a dynamic interface between the external environment and the animal’s internal cognitive map. The capacity to interpret and respond to these types of olfactory cues is fundamental to appropriately adjusting behavior, allowing animals to navigate complex environments and interact effectively with both allies and adversaries. The profound impact of olfaction on social behavior highlights its importance not just for individual survival, but also for the maintenance of ecological balances and the functioning of an entire species. In recent years, there has been growing interest in understanding the relationship between dynamic odor characteristics, their neural correlates, and behavior. This has emerged as a vibrant area of research within mammalian neuroscience [16,18–20,30,46,50–53]. Taken together, we conclude that olfaction serves as a vital entry point for detecting and decoding spatial information from sensory inputs, linking the external environment to internal cognitive processes. Building on this, we now turn to the potential underlying neural mechanisms by which complex odor dynamics are transformed across the olfactory system of mammals into perceivable patterns that guide behavior.

## Neural correlates of temporal odor dynamics

Olfactory perception starts with the binding of odor molecules to receptors located in the cilia membrane of olfactory sensory neurons (OSNs) distributed across the olfactory epithelium (OE). Due to the turbinate structure of the epithelium, the presence of a mucus layer and the subsequent signal transduction cascades, this step acts as a low-pass filter, with OSN action potentials having a rise time of approximately 80 ms for each inhalation [54]. The sniffing frequency of rodents during active sampling ranges between 2 and 15 Hz and is thereby also limited to approximately this timescale [55,56]. This is significantly slower compared to invertebrates, where OSNs have direct access to the surroundings and odor transduction can occur within 2 ms [57]. Despite such slow activation kinetics and low sampling speed, the mammalian olfactory system can still access the temporal structure of odor stimuli at unexpectedly high resolutions. These seemingly contradicting findings could be resolved by the convergence of axons from thousands of individual OSNs onto a few glomeruli in the olfactory bulb (OB), analogous to the organization of the auditory system [58], thereby preserving high-frequency odor dynamics in the OB input layer [16]. Classic go/no-go experiments with intermittency (defined as the fraction of time during which odor concentration is above a threshold in a stimulus) discrimination tasks have shown that mice can discriminate odor stimuli of low and high intermittency values [18]. It has been suggested in theoretical work that the temporal patterns of odor concentration fluctuations carry valuable spatial information about the location of odor sources [7]. Thereby, chemicals released from the same object exhibit correlated fluctuations, meaning they co-vary over time. By detecting these correlations, animals like mice can distinguish which odors come from the same source, enabling them to group related odor signals and effectively map their environment through olfactory cues. Through a systematic probing of their temporal discrimination abilities, experiments have shown that mice are indeed capable of discerning odor correlation structures at frequencies up to 40 Hz, far exceeding their sniffing rate [16]. Several groups have used an optogenetic approach to create temporal activity patterns in OSNs or the output neurons of the OB, called mitral/tufted cells (MTCs), and observe the behavioral response to these virtual odors [24,26,59–61]. These studies showed that differences in the timing of light-evoked activity of OSNs as low as 10 ms with respect to the sniff cycle [24], or the duration of activation [58], allowed mice to perform discrimination tasks. In a following study using M72-ChR2 mice, it was demonstrated that shifting the phase of the stimulus within the respiration cycle for even a single glomerulus was sufficient for discrimination [61]. In a two-choice task paradigm, during which the spatiotemporal activity pattern was varied using single or multiple-spot optogenetic stimulations, another study found that the temporal sequence of activation was indeed important in odor perception [26]. Moreover, glomeruli activated earlier in the sequence had a more substantial effect on perception than later-activated glomeruli.

**Fig 1:**
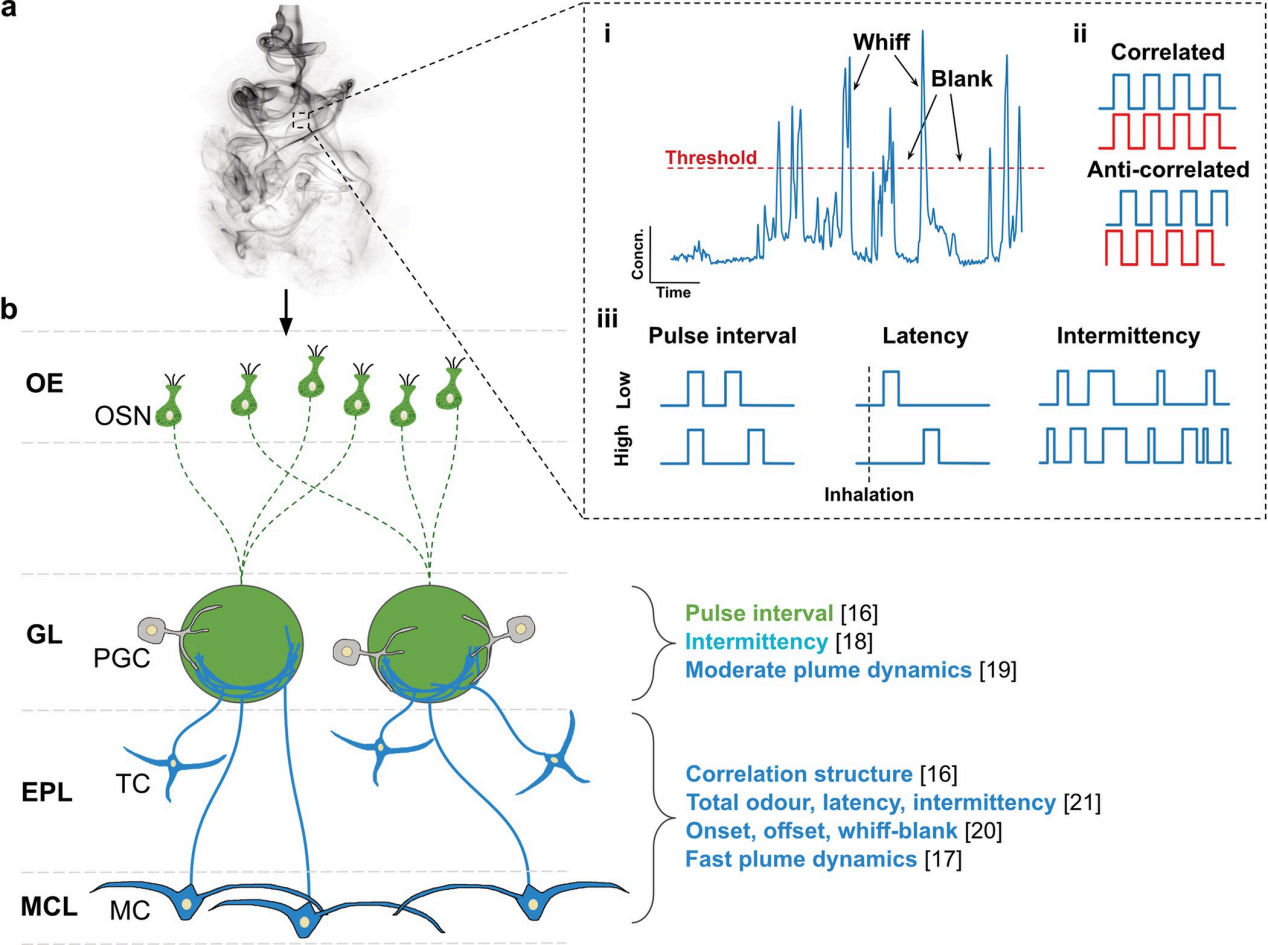
Temporal odor features represented or encoded in the olfactory bulb. **(a)** Inset: (i) Recorded concentration fluctuations in a plume showing whiffs and blanks with an arbitrary concentration threshold. (ii) Schematic representation of odor correlation structures. Blue and red pulse trains correspond to 2 different odors. (iii) Schematic representation of pulse interval, latency, and intermittency with low values in the top row and high values in the bottom row. **(b)** Olfactory bulb circuit diagram schematic depicting the layered structure and a selection of cell types involved in processing odor information. EPL, external plexiform layer; GL, glomerular layer; MC, mitral cell; MCL, mitral cell layer; OE, olfactory epithelium; OSN, olfactory sensory neuron; PGC, periglomerular cell; TC, tufted cell. The olfactory bulb circuit diagram is adapted from [62]. The plume image is adapted from (https://cr103.com/collections/Smoke/rising_smoke.jpg).

With the advent of better odor delivery and detection techniques, there is a growing interest in exploring how different temporal features are encoded and processed in the olfactory system, focusing on OSN and MTC activity patterns (Fig 1). In a recent study employing Ca^2+^ imaging in mice, odor intermittency was shown to be represented in both input and output activity in glomeruli [18]. Interestingly, responsive glomeruli clustered into 2 groups based on positive or inverse relationships between glomerular intermittency and odor intermittency. Linear classifier analyses further demonstrated that this heterogeneous population has higher prediction accuracy compared to either of the homogeneous clusters. OSN responses in the glomerular layer have also been shown to be adequate to discriminate between paired-pulse stimuli with an interval difference of 15 ms [16]. The same study revealed that MTC activity could be used to distinguish between correlated versus anti-correlated odors, while OSN responses did not faithfully encode the correlation structure. This points to the role of the local OB circuitry and perhaps centrifugal inputs in transforming OSN inputs to access temporal features at a finer temporal scale. It should be noted, however, that while stimulus dynamics could be decoded from neural activity, neither the input nor the output activity directly followed the stimulus dynamics, as also established in another study [20]. Subsequent experiments where unit recordings were performed using different odors at frequencies between 2 and 20 Hz also show congruent results that, again, while spiking profiles are not directly coupled to odor dynamics, stimulus frequency could be readily decoded from spiking activity [17]. Intriguingly, whole-cell recordings from MTCs revealed that a significant proportion of cells had distinct subthreshold membrane potential responses for 2 Hz and 20 Hz stimuli. Further studies using extracellular recordings also report that MTC response profiles have a high temporal resolution of up to 20 ms [21]. Conversely, lower-frequency features such as odor onset, offset, whiffs and blanks are directly correlated to MTC population activity [19,20], with different glomerular MTC populations exhibiting varying degrees of correlation. This correlation with large-scale temporal features was consistent with wide-field imaging of the glomerular layer [19] and high-density electrophysiological recordings from the MC and external plexiform layers [20].

Interestingly, the spiking activity recorded in this study [20] did not show any correlation with high-frequency odor fluctuations. This is likely attributed to the fact that odors used in these experiments are known to activate the dorsal portion of the OB, while recordings were mainly from the ventral OB. This might additionally suggest a timescale-dependent heterogeneity in the spatial representation of temporal features within the OB. The distribution of such heterogeneity along the dorsoventral axis for odors with different glomerular mappings would be a compelling aspect to consider in future projects. Previous studies in this direction involving simultaneous imaging of the lateral and dorsal OB or multi-electrode recordings have also reported differences in the temporal glomerular activation pattern across dorsoventral or mediolateral axes [63,64]. These differences, ascribed to the zonal distribution of OSNs in the OE and their projections to the OB [65], might provide extra temporal bandwidth for odor processing.

A different perspective to consider in this context is the sampling strategy that animals use during olfactory behaviors such as odor plume tracking or source separation. Locusts, for instance, have been shown to actively increase intermittency through antennal sweeps, possibly increasing the source localization accuracy [66]. In rodents, it is well established that a single sniff is sufficient for simple odor discrimination tasks, and, increasing the number of sniffs, for example, does not result in an equivalent increase in discrimination accuracy for monomolecular odors or binary mixtures [10,11,14]. Multiple studies have already shown that changes in sniffing frequency can alter odor responses in the OB [51,67–70] and the main olfactory cortex [71]. Varying the sniff rate can alter the temporal pattern of OSN activation and subsequent postsynaptic activity of projection neurons through both chemosensory and mechanosensory pathways [72]. Combined with differential sniff-locked activity patterns of OSNs and MTCs [24,73–75], this can potentially contribute to additional resolution in processing temporal odor dynamics.

Bilateral sampling of odors offers yet another mechanism for accessing temporal odor information. Again, to draw an example from insects, fruit flies were recently shown to use the temporal correlation of signals from the 2 antennae to perceive the direction of motion of odor [76]. It has already been established that bilateral olfactory input is essential for odor identification and localization in rodents [29,77,78] and even in humans [79,80]. This involves the anterior olfactory nucleus (AON), which is the first cortical region to integrate information from the ipsilateral and contralateral OBs and mediates most of the inter-hemispheric olfactory connections [81–83]. Different AON subregions can be symmetric or asymmetric in terms of their bilateral connectivity and show different cytoarchitectures. Although the processes underlying the integration of bilateral information are obscure, this points to a potential for comparing the temporal structure of odor stimuli, e.g., through coincidence detection. Intriguingly, a recent study demonstrated that ipsilateral and contralateral odor inputs are temporally segregated in the human piriform cortex (PCx) [84], which also receives direct input from the AON. This temporal separation implies that the brain processes odors detected by each nostril at different times, which could enhance the ability to compare and integrate sensory inputs from both sides. Since the PCx also receives direct input from the AON, which plays a role in interhemispheric communication, this highlights a potential pathway for the coordination of bilateral olfactory signals.

While there is now ample evidence to suggest that mammalian olfaction has access to complex temporal structures of olfactory stimuli even at supra-sniff frequencies, a mechanistic understanding of the local and centrifugal circuit elements involved in processing the temporal information needs to be improved. Although trial-based experimental models are necessary to do so, in natural conditions, animals are likely capable of extracting relevant information from even single sampling events [46]. Understanding such innate mechanisms calls for experimental approaches involving more naturalistic stimulus paradigms and betterment in discerning temporal features used by the circuitry. Recent developments in stimulus control and odor detection strategies, such as high-speed odor delivery devices [16] and head-mounted odor sensors [46,52], are already expediting research in these directions. With technological advancements in scanning and detection methods, faster activity sensors and large-scale electrophysiological recordings, there is immense scope in probing neural correlates of temporal odor dynamics. How these temporal features might be further translated to spatial information and used at the behavioral level is another pertinent question yet to be addressed. The OB has extensive connections to the PCx and the lateral entorhinal cortex (LEC), and the AON also receives projections from the hippocampus [81]. These regions are known to be involved in spatial information processing and encoding. In the following section, we focus on these brain areas and discuss how olfactory inputs can be integrated into spatial representations.

## How can olfactory input generate a map of the environment?

The neural substrate of cognitive maps of the environment has been largely attributed to the hippocampal formation, which is located in the medial temporal lobe, composed of the hippocampus proper (CA1-CA4 regions), dentate gyrus, subiculum, presubiculum, parasubiculum, and entorhinal cortex. The hippocampal formation plays crucial roles in memory formation, spatial navigation, and the processing of spatiotemporal information. In the CA1 layer of the hippocampus, path integration and sensory information are thought to converge, giving rise to spatially tuned neurons called place cells. Although information about various sensory modalities reaches the hippocampus [85], place cells have been primarily studied in relation to visual cues [86–88]. More recently, it has been demonstrated that olfactory information can also contribute to place field formation, stabilization, and modulation (remapping) [89–91]. This is consistent with the olfactory spatial hypothesis, which suggests a strong link between the hippocampus and olfactory areas, proposing that the evolution of the olfactory system is primarily driven by the need for navigation [38]. The anatomical and functional relationship between the hippocampus and olfactory brain regions has been established across species. In humans, performance in odor identification and spatial memory are correlated, and both can be predicted by the size of the olfactory and hippocampal areas [92]. The circuit mechanisms underlying the integration of olfactory information into the hippocampal cognitive maps, however, are not understood. It has been shown in mice that odor cues—in the absence of other sensory stimuli—gradually increase the density of place cells, and this process correlates with an improvement in navigational behavior. This supports the idea that the representation of an olfactory stimulus in CA1 is transformed into a spatial landmark [90].

**Fig 2:**
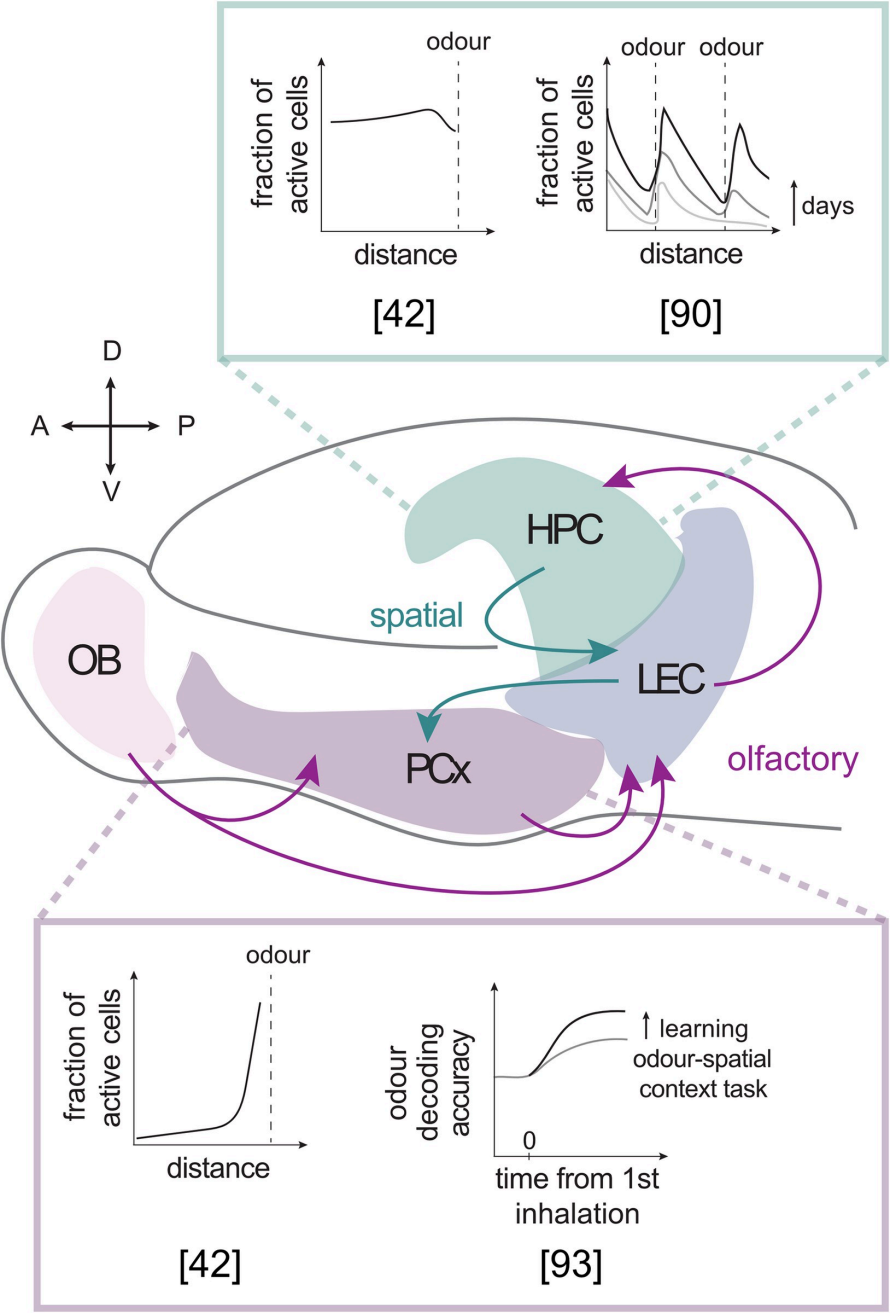
Spatial and olfactory representations in the piriform cortex and the hippocampus. Schematic illustrating the flow of olfactory and spatial information across the hippocampus-LEC-PCx axis. TOP INSERT: CA1 spatial maps provide comprehensive coverage of the environment [42]. In the absence of other sensory cues, olfactory stimuli are gradually integrated into CA1 cognitive maps [90]. BOTTOM INSERT: Place cells in the PCx correspond to odor-relevant locations [42]. Odor discrimination is enhanced after learning an odor-spatial task [93]. HPC, hippocampus; LEC, lateral entorhinal cortex; OB, olfactory bulb; PCx, piriform cortex.

It is becoming increasingly clear that spatial codes are not confined to the hippocampal formation but rather distributed throughout the entire brain (Fig 2), including areas originally thought to be primarily sensory, such as the PCx [41,42,93,94]. Information about odors and their spatial locations are thought to be encoded along a neuro-anatomical axis ranging from the PCx to the LEC, and into the hippocampus. Most recent studies have focused on the emergence of spatial maps associated with olfactory stimuli or the ability to decode spatial information from these different brain regions. However, whether the rich spatial information contained in the temporal pattern of odor plumes is translated into these maps remains unexplored. Given that the OB can decode some of the complex temporal features carried by odor plumes, it is likely that downstream regions comprising the PCx-LEC-hippocampus axis use this information for the formation of environmental maps. It will be interesting to investigate, for example, a link between responses to odor plume dynamics, such as onset latency or intermittency, and distance representation in these brain regions. Since intermittency inversely correlates with distance and has been shown to be a key feature for olfactory navigation in mice [18], exploring its relationship with the formation of spatial representations could be particularly insightful. It can be speculated that the LEC would act as an entry pathway for this information through changes in firing rate and spike timing, which could then be transmitted both to the hippocampus and the PCx. Although the field has not yet addressed these types of questions, there is a growing interest in elucidating the role of each of these regions in odor-place associations and their contribution to navigation. While it remains a subject of research, emphasizing the similarities and differences in their spatial representations may help understand how they work to form a map of the environment.

In mammals, the hippocampal formation and the PCx are evolutionarily linked and constitute a common three-layered structure known as the allocortex [95,96]. Due to similarities in their microcircuit elements, including a broad and plastic recurrent network, it has been speculated that the PCx and the hippocampus could share learning functions [97,98]. There is evidence that individual neurons in the posterior PCx of rats can encode both odor identity and spatial location when animals are engaged in a spatial task defined by odor cues [44]. This implies the formation of odor-place maps, where responses to the same odor but different spatial information are segregated. Interestingly, the accuracy of spatial information within these neurons is correlated with the strength of coherence with hippocampal theta rhythms, suggesting coordination between both structures. However, while hippocampal spatial place fields exhibit a comprehensive coverage of the space providing a continuous map of the environment, the place fields in the PCx emerge selectively in locations relevant to olfactory-driven behaviors [44]. Meanwhile, non-olfactory signals relevant to the task, including spatial information, emerge in the PCx after learning, and are associated with an increase in odor decoding accuracy (Fig 2; [93]). These results suggest that the contribution of spatial information in a primary cortex to navigational behavior may be more closely associated with enhanced sensory encoding rather than with the formation of a comprehensive spatial map of the environment.

The hippocampal circuits and the PCx are weakly connected, but they both have reciprocal connections with the LEC [99,100]. This suggests that the LEC acts as a mediator in their communication, conveying olfactory information to the hippocampal circuits and spatial context information to the PCx. The integration of LEC terminals into PCx circuits has been scarcely studied. However, it has recently been demonstrated that LEC inputs induce a reconfiguration of the PCx circuit, increasing responses to OB afferents and attenuating recurrent activity [101]. This mechanism may explain the enhancement in olfactory discrimination as a result of the integration of spatial information. This is also consistent with previous work showing that silencing LEC impairs performance on a well-learned, fine odor discrimination task [102].

The LEC plays an essential role in associative learning, allowing, for example, the association of objects with their spatial location [103–105] or the association of attributes that compose a local context [106]. Additionally, since the LEC receives a large proportion of olfactory inputs, both from the PCx and OB, it has been studied in relation to olfactory processing. A study in awake mice showed that the LEC encodes both the identity and intensity of odors and is necessary for rapid odor-guided behavior [107]. However, distinct cell types in the LEC projecting to specific brain areas are differentially involved in odor encoding, with hippocampal-projecting neurons exhibiting higher odor selectivity and better odor discrimination than PCx-projecting neurons [108]. Furthermore, it has recently been demonstrated that hippocampal-projecting neurons in the LEC are necessary for the acquisition of new odor-associative memory [109]. Yet, little research has been conducted on the contribution of the LEC to the association of odors and spatial contexts. A recent study comparing spatial and olfactory encoding in both the LEC and the PCx revealed that the PCx encodes odors more robustly, while the LEC is more accurate in encoding spatial information. Consistent with the hippocampal spatial code, the LEC shows a continuous spatial position representation, whereas the PCx predominantly represents behaviorally relevant positions [43]. In humans, it has been shown that similar spatial representations emerge in both the entorhinal and piriform cortices after learning an odor navigation task [94].

Understanding the similarities and differences in spatial and olfactory coding across the hippocampus-LEC-PCx axis is crucial for unraveling the mechanisms underlying the formation of cognitive maps based on olfactory inputs. However, further research is needed to explore how these regions process spatial information present in the complex temporal structure of natural odor plumes. Progress in this field could significantly enhance our understanding of the foundations of navigational behaviors and facilitate the development of models and technologies capable of localizing and processing dynamic odor information, which we discuss in the next section.

## Utilizing odor dynamics for technological applications

Research on artificial olfaction has led to considerable advancements in bio-inspired technologies, neural engineering, robotics, sensor technology, and computational models. Here, we highlight a series of practical implications of research into the temporal dynamics of odors, emphasizing its significant role in advancing a variety of technological domains. These innovations have wide-ranging applications, from environmental monitoring to medical diagnostics, underscoring the vast potential of integrating biological insights with technological advancements.

Artificial olfaction is commonly based on an electronic nose (eNose), a device designed to simulate the biological principles of the olfactory system [110]. eNoses often utilize arrays of chemical sensors that convert chemical signals into digital signals, enabling the device to respond to specific volatile organic compounds (VOCs). Response times of conventional metal-oxide (MOx) gas sensors lie in the range of minutes [111]. Recent hardware and software advancements of MOx gas sensor arrays have given rise to a new generation of sensors that detect VOCs with unprecedented response times at second or even sub-second precision [112–116]. A newly developed miniaturized high-speed MOx gas sensor can classify odor pulses with a temporal resolution of only tens of milliseconds and encode stimuli switching up to 60 Hz [113]. This is a previously unmatched timescale which even exceeds the odor correlation discrimination performance observed in mice [16]. These developments have dramatically improved the ability to detect and process odor dynamics swiftly, mimicking the response to specific VOCs observed in biological olfactory systems. Artificial olfactory sensors have been beneficial in numerous applications, including environmental monitoring, where eNoses are used to detect hazardous gases such as carbon monoxide and hydrocarbons, as well as to identify pollutants like benzene, toluene, and xylene in the atmosphere [117]. eNoses equipped with arrays of chemical sensors have the ability to detect and differentiate between complex odor mixtures. These sensors respond to various chemical compounds by producing a pattern or “signature” that is specific to each detected compound [118]. This feature becomes essential when monitoring environments where sudden increases in toxic gases or pollutants could have detrimental effects on health and safety. The ability of sensors to extract rapid odor fluctuations with high sensitivity [113,116] will help to develop novel strategies for robotic noses that mimic the advanced olfactory processing capabilities of biological systems. Unlike traditional detectors that focus on merely identifying specific target molecules, the biological olfactory system is able to interpret complex temporal and spatial patterns in odor plumes to navigate and make decisions. By integrating fast odor sensors capable of capturing millisecond-scale plume dynamics, robotic noses could process these temporal patterns to distinguish between different odor sources, determine the direction and distance of an odor, and adapt to dynamic environments. This approach moves beyond detection, enabling robots to utilize odor cues for navigation, search and rescue operations, environmental monitoring, and industrial applications where understanding the temporal dynamics of odor dispersion is crucial.

Mobile olfactory robots provide the opportunity to handle complex tasks, such as navigating towards or away from specific odors based on programmed objectives [119–121]. This can serve as an invaluable asset to approach or scan potentially hazardous environments, for example, during environmental monitoring or search and rescue operations. An application becomes particularly attractive when robots are equipped with high-precision, durable sensors and sophisticated algorithms for data processing and interpretation. A challenge, however, remains the real-time interpretation of odor signals with high temporal resolution that incorporates environmental interference and odor signal overlap. Given the rapid advancements in multimodal signal detection, integration, and processing, as well as the kinetic capabilities of modern robots, we can expect to see more sophisticated, efficient, and autonomous robotic systems capable of leveraging olfactory data for a wide range of tasks.

Despite the strides made in sensor technology, the challenge of accurately identifying odors at an appropriate temporal resolution to harness the information carried by odor dynamics remains a significant hurdle. To overcome this, research is focused on understanding the mechanisms behind olfactory recognition and applying engineering principles to replicate these processes [122]. To this end, artificial recurrent network agents trained to locate the source of simulated odor plumes allow the prediction of memory requirements and the neural dynamics underlying this behavior [123]. The development of olfactory brain–computer interfaces provides another promising approach to bridge biology and technology through a more profound understanding of odor processing [124]. These innovations not only enhance our interaction with the natural world but also open up new avenues for technological integration and application across diverse fields.

## Conclusions

Research on the spatiotemporal aspect of olfactory information has gained considerable traction over the last few years. Despite significant advances in odor delivery and large-scale recording techniques, there remains substantial scope for further research, particularly in understanding the finer aspects and mechanisms of neural processing in the olfactory bulb and higher cortical areas. Future studies are likely to delve deeper into how odor-evoked neural activity induces behavioral outcomes in more dynamic settings. Combining more naturalistic behavioral assays with neurophysiological recordings using high-density silicon probes and the latest jGCaMP8 calcium indicators [125] will further help to identify the neural circuits that integrate temporal odor information with spatial memory. Furthermore, complementary computational modeling and machine learning techniques that simulate rodent navigation strategies will allow us to predict how different cues contribute to their behavior.

The integration of olfactory sensors in technology, particularly in robotics and real-time monitoring systems, presents an exciting frontier. These developments hold the promise of enhancing the sensitivity and adaptability of automated systems, potentially mirroring the nuanced perceptual capabilities found in nature. We conclude that olfaction offers a unique gateway to mechanistically understand how spatial information reaches and gets processed in the brain. By continuing to explore these areas, we can anticipate not only a richer understanding of olfactory neuroscience but also significant advancements in the practical applications of this knowledge.

## Abbreviations

AON: anterior olfactory nucleus
LEC: lateral entorhinal cortex
MTC: mitral/tufted cell
OB: olfactory bulb
OE: olfactory epithelium
OSN: olfactory sensory neuron
PCx: piriform cortex; VOC, volatile organic compound

## References

[1] Mori K, Nagao H, Yoshihara Y. The olfactory bulb: Coding and processing of odor molecule information. Science. 1999;286(5440):711–5. doi: 10.1126/science.286.5440.711 10531048

[2] Moore P, Crimaldi J. Odor landscapes and animal behavior: Tracking odor plumes in different physical worlds. J Mar Syst. 2004;49(1–4):55–64. doi: 10.1016/j.jmarsys.2003.05.005

[3] Murlis J, Elkington JS, Carde RT. Odor Plumes And How Insects Use Them. Annu Rev Entomol. 1992;37(1):505–32. doi: 10.1146/annurev.en.37.010192.002445

[4] Mylne KR, Mason PJ. Concentration fluctuation measurements in a dispersing plume at a range of up to 1000m. Q J Roy Meteorol Soc. 1991 Jan 1;117(497):177–206. doi: 10.1002/qj.49711749709

[5] Shraiman BI, Siggia ED, Shralman BI, Siggia ED. Scalar turbulence. Nature. 2000 Jun 8;405(6787):639. doi: 10.1038/35015000 10864314

[6] Celani A, Villermaux E, Vergassola M. Odor Landscapes in Turbulent Environments. Phys Rev X. 2014;4(4):041015. doi: 10.1103/PhysRevX.4.041015

[7] Hopfield JJ. Olfactory computation and object perception. Proc Natl Acad Sci U S A. 1991 Aug 1;88(15):6462–6. doi: 10.1073/pnas.88.15.6462 1862075 PMC52105

[8] Murlis J, Willis MA, Cardé RT. Spatial and temporal structures of pheromone plumes in fields and forests. Physiol Entomol. 2000;25(3):211–22. doi: 10.1046/j.1365-3032.2000.00176.x

[9] Reddy G, Murthy VN, Vergassola M. Olfactory Sensing and Navigation in Turbulent Environments. Annu Rev Condens Matter Phys. 2022;13(1):191–213. doi: 10.1146/annurev-conmatphys-031720-032754 PMC12758385 41488225

[10] Abraham NM, Spors H, Carleton A, Margrie TW, Kuner T, Schaefer AT. Maintaining accuracy at the expense of speed: Stimulus similarity defines odor discrimination time in mice. Neuron. 2004;44:865–76. doi: 10.1016/j.neuron.2004.11.017 15572116

[11] Rinberg D, Koulakov A, Gelperin A. Speed-Accuracy Tradeoff in Olfaction. Neuron 2006 Aug 3;51(3):351–8. doi: 10.1016/j.neuron.2006.07.013 16880129

[12] Slotnick B. Odor-Sampling Time of Mice under Different Conditions. Chem Senses. 2007 Jun 1;32(5):445–54. doi: 10.1093/chemse/bjm013 17426048

[13] Laing DG. Identification of single dissimilar odors is achieved by humans with a single sniff. Physiol Behav. 1986 Jan 1;37(1):163–70. doi: 10.1016/0031-9384(86)90400-2 3737714

[14] Uchida N, Mainen ZF. Speed and accuracy of olfactory discrimination in the rat. Nat Neurosci. 2003 Nov;6(11):1224–9. doi: 10.1038/nn1142 14566341

[15] Wesson DW, Carey RM, Verhagen JV, Wachowiak M. Rapid encoding and perception of novel odors in the rat. PLoS Biol. 2008;6(4):717–29. doi: 10.1371/journal.pbio.0060082 18399719 PMC2288628

[16] Ackels T, Erskine A, Dasgupta D, Marin AC, Warner TPA, Tootoonian S, et al. Fast odour dynamics are encoded in the olfactory system and guide behaviour. Nature. 2021 May 27;593(7860):558–63. doi: 10.1038/s41586-021-03514-2 33953395 PMC7611658

[17] Dasgupta D, Warner TPA, Erskine A, Schaefer AT. Coupling of mouse olfactory bulb projection neurons to fluctuating odour pulses. J Neurosci. 2022 Apr 19;JN-RM-1422–21. doi: 10.1523/jneurosci.1422-21.2022 35440491 PMC9145232

[18] Gumaste A, Baker KL, Izydorczak M, True AC, Vasan G, Crimaldi JP, et al. Behavioral discrimination and olfactory bulb encoding of odor plume intermittency. Pírez N, Sengupta P, editors. Elife. 2024 Mar 5;13:e85303. doi: 10.7554/eLife.85303 38441541 PMC11001298

[19] Lewis SM, Xu L, Rigolli N, Tariq MF, Suarez LM, Stern M, et al. Plume Dynamics Structure the Spatiotemporal Activity of Mitral/Tufted Cell Networks in the Mouse Olfactory Bulb. Front Cell Neurosci [Internet]. 2021 Apr 30;15. Available from: https://www.frontiersin.org/articles/10.3389/fncel.2021.633757. doi: 10.3389/fncel.2021.633757 34012385 PMC8127944

[20] Lewis SM, Suarez LM, Rigolli N, Steinmetz NA, Gire DH. The spiking output of the mouse olfactory bulb encodes large-scale temporal features of natural odor environments [Internet]. 2024. Available from: http://biorxiv.org/lookup/doi/ doi: 10.1101/2024.03.01.582978 38496526 PMC10942328

[21] Warner TPA, Tootoonian S, Schaefer AT. Robust encoding of sub-sniff temporal information in the mouse olfactory bulb [Internet]. bioRxiv. 2024. p. 2024.03.26.586830. Available from: https://www.biorxiv.org/content/10.1101/2024.03.26.586830v2.

[22] Demir M, Kadakia N, Anderson HD, Clark DA, Emonet T. Walking Drosophila navigate complex plumes using stochastic decisions biased by the timing of odor encounters. Seminara A, Calabrese RL, Murthy VN, Celani A, editors. Elife. 2020 Nov 3;9:e57524. doi: 10.7554/eLife.57524 33140723 PMC7609052

[23] Stierle JS, Galizia CG, Szyszka P. Millisecond stimulus onset-asynchrony enhances information about components in an odor mixture. J Neurosci. 2013 Apr 3;33(14):6060–9. doi: 10.1523/JNEUROSCI.5838-12.2013 23554487 PMC6618935

[24] Smear M, Shusterman R, O’Connor R, Bozza T, Rinberg D. Perception of sniff phase in mouse olfaction. Nature. 2011 Nov 17;479(7373):397–400. doi: 10.1038/nature10521 21993623

[25] Wilson CD, Serrano GO, Koulakov AA, Rinberg D. A primacy code for odor identity. Nat Commun. 2017;8(1):1477. doi: 10.1038/s41467-017-01432-4 29133907 PMC5684307

[26] Chong E, Moroni M, Wilson C, Shoham S, Panzeri S, Rinberg D. Manipulating synthetic optogenetic odors reveals the coding logic of olfactory perception. Science. 2020 Jun 19;368(6497):eaba2357. doi: 10.1126/science.aba2357 32554567 PMC8237706

[27] Arakawa H, Arakawa K, Blanchard DC, Blanchard RJ. A new test paradigm for social recognition evidenced by urinary scent marking behavior in C57BL/6J mice. Behav Brain Res. 2008 Jun;190(1):97–104. doi: 10.1016/j.bbr.2008.02.009 18359521 PMC2441767

[28] Hepper PG, Wells DL. How Many Footsteps Do Dogs Need to Determine the Direction of an Odour Trail? Chem Senses. 2005 May 1;30(4):291–8. doi: 10.1093/chemse/bji023 15741595

[29] Catania KC. Stereo and serial sniffing guide navigation to an odour source in a mammal. Nat Commun. 2013;4:1441. doi: 10.1038/ncomms2444 23385586

[30] Findley TM, Wyrick DG, Cramer JL, Brown MA, Holcomb B, Attey R, et al. Sniff-synchronized, gradient-guided olfactory search by freely moving mice. Elife. 2021;10:1–39. doi: 10.7554/eLife.58523 33942713 PMC8169121

[31] Jones PW, Urban NN. Mice follow odor trails using stereo olfactory cues and rapid sniff to sniff comparisons [Internet]. bioRxiv. 2018. p. 293746. Available from: https://www.biorxiv.org/content/10.1101/293746v2.

[32] Khan AG, Sarangi M, Bhalla US. Rats track odour trails accurately using a multi-layered strategy with near-optimal sampling. Nat Commun. 2012;3:703. doi: 10.1038/ncomms1712 22426224

[33] Porter J, Craven B, Khan RM, Chang SJ, Kang I, Judkewitz B, et al. Mechanisms of scent-tracking in humans. Nat Neurosci. 2007;10(1):27–9. doi: 10.1038/nn1819 17173046

[34] Reddy G, Shraiman BI, Vergassola M. Sector search strategies for odor trail tracking. Proc Natl Acad Sci U S A. 2022;119(1):1–8. doi: 10.1073/pnas.2107431118 34983837 PMC8740577

[35] Jinn J, Connor EG, Jacobs LF. How Ambient Environment Influences Olfactory Orientation in Search and Rescue Dogs. Chem Senses. 2020 Nov 7;45(8):625–34. doi: 10.1093/chemse/bjaa060 32940645

[36] Howard WE, Marsh RE, Cole RE. Food detection by deer mice using olfactory rather than visual cues. Anim Behav. 1968;16(1):13–7. doi: 10.1016/0003-3472(68)90100-0 5639893

[37] Crimaldi J, Lei H, Schaefer A, Schmuker M, Smith BH, True AC, et al. Active sensing in a dynamic olfactory world. J Comput Neurosci [Internet]. 2021. doi: 10.1007/s10827-021-00798-1 34591220

[38] Jacobs LF. From chemotaxis to the cognitive map: The function of olfaction. Proc Natl Acad Sci U S A. 2012;109:10693–700. doi: 10.1073/pnas.1201880109 22723365 PMC3386877

[39] Boie SD, Connor EG, McHugh M, Nagel KI, Ermentrout GB, Crimaldi JP, et al. Information-theoretic analysis of realistic odor plumes: What cues are useful for determining location? PLoS Comput Biol. 2018;14(7):1–19. doi: 10.1371/journal.pcbi.1006275 29990365 PMC6054425

[40] Victor JD, Boie SD, Connor EG, Crimaldi JP, Ermentrout GB, Nagel KI. Olfactory navigation and the receptor nonlinearity. J Neurosci. 2019;39(19):3713–27. doi: 10.1523/JNEUROSCI.2512-18.2019 30846614 PMC6510337

[41] Mena W, Baker K, Rubin A, Kohli S, Yoo Y, Ziv Y, et al. Differential encoding of odor and place in mouse piriform and entorhinal cortex [Internet]. bioRxiv. 2023. p. 2023.10.05.561119. Available from: https://www.biorxiv.org/content/10.1101/2023.10.05.561119v1 10.1523/ENEURO.0026-25.2025 PMC12507510 40973488

[42] Poo C, Agarwal G, Bonacchi N, Mainen ZF. Spatial maps in piriform cortex during olfactory navigation. Nature. 2022 Jan;601(7894):595–9. doi: 10.1038/s41586-021-04242-3 34937941

[43] Gire DH, Kapoor V, Arrighi-Allisan A, Seminara A, Murthy VN. Mice Develop Efficient Strategies for Foraging and Navigation Using Complex Natural Stimuli. Curr Biol. 2016 May;26(10):1261–73. doi: 10.1016/j.cub.2016.03.040 27112299 PMC4951102

[44] Jacobs LF, Arter J, Cook A, Sulloway FJ. Olfactory Orientation and Navigation in Humans. PLoS ONE. 2015 Jun 17;10(6):e0129387. doi: 10.1371/journal.pone.0129387 26083337 PMC4470656

[45] Perl O, Nahum N, Belelovsky K, Haddad R. The contribution of temporal coding to odor coding and odor perception in humans. eLife. 9:e49734. doi: 10.7554/eLife.49734 32031520 PMC7007219

[46] Wu Y, Chen K, Xing C, Huang M, Zhao K, Zhou W. Human olfactory perception embeds fine temporal resolution within a single sniff. Nat Hum Behav [Internet]. 2024 Oct 14. Available from: https://www.nature.com/articles/s41562-024-01984-8. doi: 10.1038/s41562-024-01984-8 39402256

[47] Liberles SD. Mammalian Pheromones. Annu Rev Physiol. 2014 Aug 26;76(1):151–75. doi: 10.1146/annurev-physiol-021113-170334 23988175 PMC4310675

[48] Wachowiak M. All in a Sniff: Olfaction as a Model for Active Sensing. Neuron. 2011 Sep 22;71(6):962–73. doi: 10.1016/j.neuron.2011.08.030 21943596 PMC3237116

[49] Stowers L, Liberles SD. State-dependent responses to sex pheromones in mouse. Curr Opin Neurobiol. 2016;38:74–9. doi: 10.1016/j.conb.2016.04.001 27093585 PMC4921285

[50] Gumaste A, Coronas-Samano G, Hengenius J, Axman R, Connor EG, Baker KLK, et al. A Comparison between Mouse, In Silico, and Robot Odor Plume Navigation Reveals Advantages of Mouse Odor Tracking. eNeuro. 2020 Jan;7(1):ENEURO.0212-19.2019. doi: 10.1523/ENEURO.0212-19.2019 31924732 PMC7004486

[51] Jordan R, Kollo M, Schaefer AT. Sniffing Fast: Paradoxical Effects on Odor Concentration Discrimination at the Levels of Olfactory Bulb Output and Behavior. eNeuro. 2018 Sep;5(5):ENEURO.0148-18.2018. doi: 10.1523/ENEURO.0148-18.2018 30596145 PMC6306510

[52] Tariq MF, Lewis SM, Lowell A, Moore S, Miles JT, Perkel DJ, et al. Using Head-Mounted Ethanol Sensors to Monitor Olfactory Information and Determine Behavioral Changes Associated with Ethanol-Plume Contact during Mouse Odor-Guided Navigation. eNeuro. 2021 Jan 8;8(1):ENEURO.0285-20.2020. doi: 10.1523/ENEURO.0285-20.2020 33419862 PMC7877453

[53] Tariq MF, Sterrett SC, Moore S, Lane, Perkel DJ, Gire DH. Dynamics of odor-source localization: Insights from real-time odor plume recordings and head-motion tracking in freely moving mice. PLoS ONE. 2024 Sep 26;19(9):e0310254. doi: 10.1371/journal.pone.0310254 39325742 PMC11426488

[54] Carey RM, Verhagen JV, Wesson DW, Pírez N, Wachowiak M. Temporal Structure of Receptor Neuron Input to the Olfactory Bulb Imaged in Behaving Rats. J Neurophysiol. 2009 Feb;101(December 2008):1073–88. doi: 10.1152/jn.90902.2008 19091924 PMC2657066

[55] Kepecs A, Uchida N, Mainen ZF. The sniff as a unit of olfactory processing. Chem Senses. 2006;31(2):167–79. doi: 10.1093/chemse/bjj016 16339265

[56] Welker WI. Analysis of Sniffing of the Albino Rat. Behaviour. 1964;22(3):223–44. https://psycnet.apa.org/doi/10.1163/156853964X00030

[57] Szyszka P, Gerkin RC, Galizia CG, Smith BH. High-speed odor transduction and pulse tracking by insect olfactory receptor neurons. Proc Natl Acad Sci U S A. 2014 Nov 25;111(47):16925–30. doi: 10.1073/pnas.1412051111 25385618 PMC4250155

[58] Carr CE, Amagai S. Processing of temporal information in the brain. Adv Psychol. 1996;115(C):27–52. doi: 10.1146/annurev.ne.16.030193.001255 8460892

[59] Li A, Gire DH, Bozza T, Restrepo D. Precise Detection of Direct Glomerular Input Duration by the Olfactory Bulb. J Neurosci. 2014 Nov 26;34(48):16058–64. doi: 10.1523/JNEUROSCI.3382-14.2014 25429146 PMC4244471

[60] Rebello MR, McTavish TS, Willhite DC, Short SM, Shepherd GM, Verhagen JV. Perception of Odors Linked to Precise Timing in the Olfactory System. PLoS Biol. 2014 Dec 16;12(12):e1002021. doi: 10.1371/journal.pbio.1002021 25514030 PMC4267717

[61] Smear M, Resulaj A, Zhang J, Bozza T, Rinberg D. Multiple perceptible signals from a single olfactory glomerulus. Nat Neurosci. 2013 Nov 22;2013(11):1687–91. doi: 10.1038/nn.3519 24056698 PMC13445371

[62] Nagayama S, Homma R, Imamura F. Neuronal organization of olfactory bulb circuits. Front Neural Circuits. 2014 Sep 3;8(September):1–19. doi: 10.3389/fncir.2014.00098 25232305 PMC4153298

[63] Baker KL, Vasan G, Gumaste A, Pieribone VA, Verhagen JV. Spatiotemporal dynamics of odor responses in the lateral and dorsal olfactory bulb. PLoS Biol. 2019;17(9):1–23. doi: 10.1371/journal.pbio.3000409 31532763 PMC6768483

[64] Zhou Z, Belluscio L. Coding Odorant Concentration through Activation Timing between the Medial and Lateral Olfactory Bulb. Cell Rep. 2012 Nov 29;2(5):1143–50. doi: 10.1016/j.celrep.2012.09.035 23168258 PMC3513620

[65] Miyamichi K, Serizawa S, Kimura HM, Sakano H. Continuous and Overlapping Expression Domains of Odorant Receptor Genes in the Olfactory Epithelium Determine the Dorsal/Ventral Positioning of Glomeruli in the Olfactory Bulb. J Neurosci. 2005 Apr 6;25(14):3586–92. doi: 10.1523/JNEUROSCI.0324-05.2005 15814789 PMC6725380

[66] Huston SJ, Stopfer M, Cassenaer S, Aldworth ZN, Laurent G. Neural Encoding of Odors during Active Sampling and in Turbulent Plumes. Neuron. 2015 Oct;88(2):403–18. doi: 10.1016/j.neuron.2015.09.007 26456047 PMC4742573

[67] Díaz-quesada M, Youngstrom IA, Tsuno XY, Hansen XKR, Economo MN, Wachowiak XM, et al. Inhalation frequency controls reformatting of mitral/tufted cell odor representations in the olfactory bulb. J Neurosci. 2018;38(9):2189–206. doi: 10.1523/JNEUROSCI.0714-17.2018 29374137 PMC5830510

[68] Jordan R, Fukunaga I, Kollo M, Schaefer AT. Active Sampling State Dynamically Enhances Olfactory Bulb Odor Representation. Neuron. 2018 Jun;98(6):1214–28. doi: 10.1016/j.neuron.2018.05.016 29861286 PMC6030445

[69] Verhagen JV, Wesson DW, Netoff TI, White JA, Wachowiak M. Sniffing controls an adaptive filter of sensory input to the olfactory bulb. Nat Neurosci. 2007 May 22;10(5):631–9. doi: 10.1038/nn1892 17450136

[70] Shusterman R, Sirotin YB, Smear MC, Ahmadian Y, Rinberg D. Sniff Invariant Odor Coding. eNeuro. 2018 Nov;5(6):ENEURO.0149-18.2018. doi: 10.1523/ENEURO.0149-18.2018 30627641 PMC6325545

[71] Dehaqani AA, Michelon F, Patella P, Petrucco L, Piasini E, Iurilli G. A mechanosensory feedback that uncouples external and self-generated sensory responses in the olfactory cortex. Cell Rep. 2024 Apr 23;43(4). doi: 10.1016/j.celrep.2024.114013 38551962

[72] Grosmaitre X, Santarelli LC, Tan J, Luo M, Ma M. Dual functions of mammalian olfactory sensory neurons as odor detectors and mechanical sensors. Nat Neurosci. 2007;10(3):348–54. doi: 10.1038/nn1856 17310245 PMC2227320

[73] Ackels T, Jordan R, Schaefer AT, Fukunaga I. Respiration-Locking of Olfactory Receptor and Projection Neurons in the Mouse Olfactory Bulb and Its Modulation by Brain State. Front Cell Neurosci. 2020;14(July):1–15. doi: 10.3389/fncel.2020.00220 32765224 PMC7378796

[74] Iwata R, Kiyonari H, Imai T. Mechanosensory-Based Phase Coding of Odor Identity in the Olfactory Bulb. Neuron. 2017 Dec;96(5):1139–52. doi: 10.1016/j.neuron.2017.11.008 29216451

[75] Shusterman R, Smear MC, Koulakov AA, Rinberg D. Precise olfactory responses tile the sniff cycle. Nat Neurosci. 2011 Aug;14(8):1039–44. doi: 10.1038/nn.2877 21765422 PMC13348895

[76] Kadakia N, Demir M, Michaelis BT, DeAngelis BD, Reidenbach MA, Clark DA, et al. Odour motion sensing enhances navigation of complex plumes. Nature 2022 Nov;611(7937):754–61. doi: 10.1038/s41586-022-05423-4 36352224 PMC10039482

[77] Esquivelzeta Rabell J, Mutlu K, Noutel J, Martin del Olmo P, Haesler S. Spontaneous Rapid Odor Source Localization Behavior Requires Interhemispheric Communication. Curr Biol. 2017 May 22;27(10):1542–1548.e4. doi: 10.1016/j.cub.2017.04.027 28502658

[78] Kuruppath P, Bai L, Belluscio L. Functional Relevance of Dual Olfactory Bulbs in Olfactory Coding. eNeuro. 2021 Sep;8(5):ENEURO.0070-21.2021. doi: 10.1523/ENEURO.0070-21.2021 34413085 PMC8422849

[79] Welge-Lussen A, Looser GL, Westermann B, Hummel T. Olfactory source localization in the open field using one or both nostrils. Rhinology. 2014 Mar;52(1):41–7. doi: 10.4193/Rhino13.108 24618627

[80] Wu Y, Chen K, Ye Y, Zhang T, Zhou W. Humans navigate with stereo olfaction. Proc Natl Acad Sci U S A. 2020 Jul 7;117(27):16065–71. doi: 10.1073/pnas.2004642117 32571945 PMC7355004

[81] Brunert D, Quintela RM, Rothermel M. The anterior olfactory nucleus revisited–an emerging role for neuropathological conditions? Prog Neurobiol. 2023 Jun 19;102486. doi: 10.1016/j.pneurobio.2023.102486 37343762

[82] Dalal T, Gupta N, Haddad R. Bilateral and unilateral odor processing and odor perception. Commun Biol. 2020 Dec 1;3(1):150. doi: 10.1038/s42003-020-0876-6 32238904 PMC7113286

[83] Kikuta S, Sato K, Kashiwadani H, Tsunoda K, Yamasoba T, Mori K. Neurons in the anterior olfactory nucleus pars externa detect right or left localization of odor sources. Proc Natl Acad Sci U S A. 2010 Jul 6;107(27):12363–8. doi: 10.1073/pnas.1003999107 20616091 PMC2901466

[84] Dikeçligil GN, Yang AI, Sanghani N, Lucas T, Chen HI, Davis KA, et al. Odor representations from the two nostrils are temporally segregated in human piriform cortex. Curr Biol. 2023 Dec 18;33(24):5275–5287.e5. doi: 10.1016/j.cub.2023.10.021 37924807 PMC13035057

[85] Ranck JB. Studies on single neurons in dorsal hippocampal formation and septum in unrestrained rats: Part I. Behavioral correlates and firing repertoires. Exp Neurol. 1973 Nov 1;41(2):462–531 doi: 10.1016/0014-4886(73)90290-2 . 4355646

[86] Cressant A, Muller RU, Poucet B. Failure of Centrally Placed Objects to Control the Firing Fields of Hippocampal Place Cells. J Neurosci. 1997 Apr 1;17(7):2531–42. doi: 10.1523/JNEUROSCI.17-07-02531.1997 9065513 PMC6573492

[87] Muller RU, Kubie JL. The effects of changes in the environment on the spatial firing of hippocampal complex-spike cells. J Neurosci. 1987 Jul 1;7(7):1951–68. doi: 10.1523/JNEUROSCI.07-07-01951.1987 3612226 PMC6568940

[88] O’Keefe J, Conway DH. Hippocampal place units in the freely moving rat: Why they fire where they fire. Exp Brain Res. 1978 Apr 1;31(4):573–90. doi: 10.1007/BF00239813 658182

[89] Anderson MI, Jeffery KJ. Heterogeneous Modulation of Place Cell Firing by Changes in Context. J Neurosci 2003 Oct 1;23(26):8827–35. doi: 10.1523/JNEUROSCI.23-26-08827.2003 14523083 PMC6740394

[90] Fischler-Ruiz W, Clark D, Joshi N, Devi-Chou V, Kitch L, Schnitzer MJ, et al. Olfactory Landmarks and Path Integration Converge to Form a Cognitive Spatial Map. Neuron. 2021;1–14. doi: 10.1016/j.neuron.2021.09.055 34710366

[91] Zhang S, Manahan-Vaughan D. Spatial Olfactory Learning Contributes to Place Field Formation in the Hippocampus. Cereb Cortex. 2015 Feb 1;25(2):423–32. doi: 10.1093/cercor/bht239 24008582 PMC4380081

[92] Dahmani L, Patel RM, Yang Y, Chakravarty MM, Fellows LK, Bohbot VD. An intrinsic association between olfactory identification and spatial memory in humans. Nat Commun. 2018 Oct 16;9(1):4162. doi: 10.1038/s41467-018-06569-4 30327469 PMC6191417

[93] Federman N, Romano SA, Amigo-Duran M, Salomon L, Marin-Burgin A. Acquisition of non-olfactory encoding improves odour discrimination in olfactory cortex. Nat Commun. 2024 Jul 2;15(1):5572. doi: 10.1038/s41467-024-49897-4 38956072 PMC11220071

[94] Raithel CU, Miller AJ, Epstein RA, Kahnt T, Gottfried JA. Recruitment of grid-like responses in human entorhinal and piriform cortices by odor landmark-based navigation. Curr Biol. 2023 Sep 11;33(17):3561–3570.e4. doi: 10.1016/j.cub.2023.06.087 37506703 PMC10510564

[95] Klingler E. Development and Organization of the Evolutionarily Conserved Three-Layered Olfactory Cortex. eNeuro [Internet]. 2017 Jan 1;4(1). Available from: doi: 10.1523/ENEURO.0193-16.2016 28144624 PMC5272922

[96] Shepherd GM. The Microcircuit Concept Applied to Cortical Evolution: from Three-Layer to Six-Layer Cortex. Front Neuroanat 2011 May 23;5. doi: 10.3389/fnana.2011.00030 21647397 PMC3102215

[97] Barkai E, Hasselmo MH. Acetylcholine and associative memory in the piriform cortex. Mol Neurobiol. 1997 Aug 1;15(1):17–29. doi: 10.1007/BF02740613 9396002

[98] Johnson DMG, Illig KR, Behan M, Haberly LB. New Features of Connectivity in Piriform Cortex Visualized by Intracellular Injection of Pyramidal Cells Suggest that “Primary” Olfactory Cortex Functions Like “Association” Cortex in Other Sensory Systems. J Neurosci. 2000 Sep 15;20(18):6974–82. doi: 10.1523/JNEUROSCI.20-18-06974.2000 10995842 PMC6772836

[99] Agster KL, Burwell RD. Cortical efferents of the perirhinal, postrhinal, and entorhinal cortices of the rat. Hippocampus 2009;19(12):1159–86. doi: 10.1002/hipo.20578 19360714 PMC3066185

[100] Witter MP, Doan TP, Jacobsen B, Nilssen ES, Ohara S. Architecture of the Entorhinal Cortex A Review of Entorhinal Anatomy in Rodents with Some Comparative Notes. Front Syst Neurosci [Internet]. 2017 Jun 28 [cited 2024 Sep 23];11. Available from: https://www.frontiersin.org/journals/systems-neuroscience/articles/10.3389/fnsys.2017.00046 28701931 10.3389/fnsys.2017.00046 PMC5488372

[101] Pedroncini O, Federman N, Marin-Burgin A. Lateral entorhinal cortex afferents reconfigure the activity in piriform cortex circuits [Internet]. bioRxiv. 2024. p. 2024.06.16.599205. Available from: https://www.biorxiv.org/content/10.1101/2024.06.16.599205v1. 10.1073/pnas.2414038121 PMC11621770 39570314

[102] Chapuis J, Cohen Y, He X, Zhang Z, Jin S, Xu F, et al. Lateral entorhinal modulation of piriform cortical activity and fine odor discrimination. J Neurosci 2013;33(33):13449–59. doi: 10.1523/JNEUROSCI.1387-13.2013 23946403 PMC3742931

[103] Beer Z, Chwiesko C, Kitsukawa T, Sauvage MM. Spatial and stimulus-type tuning in the LEC, MEC, POR, PrC, CA1, and CA3 during spontaneous item recognition memory. Hippocampus. 2013;23(12):1425–38. doi: 10.1002/hipo.22195 23966131

[104] Chao OY, Huston JP, Li JS, Wang AL, de Souza Silva MA. The medial prefrontal cortex—lateral entorhinal cortex circuit is essential for episodic-like memory and associative object-recognition. Hippocampus. 2016;26(5):633–45. doi: 10.1002/hipo.22547 26501829

[105] Wilson DIG, Langston RF, Schlesiger MI, Wagner M, Watanabe S, Ainge JA. Lateral entorhinal cortex is critical for novel object-context recognition. Hippocampus. 2013;23(5):352–66. doi: 10.1002/hipo.22095 23389958 PMC3648979

[106] Kuruvilla MV, Ainge JA. Lateral Entorhinal Cortex Lesions Impair Local Spatial Frameworks. Front Syst Neurosci. 2017 May 17;11. doi: 10.3389/fnsys.2017.00030 28567006 PMC5434111

[107] Bitzenhofer SH, Westeinde EA, Zhang HXB, Isaacson JS. Rapid odor processing by layer 2 subcircuits in lateral entorhinal cortex. Elife. 2022 Feb 7;11. doi: 10.7554/eLife.75065 35129439 PMC8860446

[108] Leitner FC, Melzer S, Lütcke H, Pinna R, Seeburg PH, Helmchen F, et al. Spatially segregated feedforward and feedback neurons support differential odor processing in the lateral entorhinal cortex. Nat Neurosci. 2016 Jul 16;19(7):935–44. doi: 10.1038/nn.4303 27182817

[109] Lee JY, Jun H, Soma S, Nakazono T, Shiraiwa K, Dasgupta A, et al. Dopamine facilitates associative memory encoding in the entorhinal cortex. Nature. 2021 Oct;598(7880):321–6. doi: 10.1038/s41586-021-03948-8 34552245 PMC8744500

[110] Covington JA, Marco S, Persaud KC, Schiffman SS, Nagle HT. Artificial Olfaction in the 21st Century. IEEE Sens J. 2021 Jun;21(11):12969–90. doi: 10.1109/JSEN.2021.3076412

[111] Pashami S, Lilienthal A, Trincavelli M. Detecting Changes of a Distant Gas Source with an Array of MOX Gas Sensors. Sensors. 2012 Nov 27;12(12):16404–19. doi: 10.3390/s121216404 23443385 PMC3571789

[112] Burgués J, Valdez L, Marco S. High-bandwidth e-nose for rapid tracking of turbulent plumes. 2019. 1 p. doi: 10.1109/ISOEN.2019.8823158

[113] Dennler N, Drix D, Warner TPA, Rastogi S, Della Casa C, Ackels T, et al. High-speed odour sensing using miniaturised electronic nose. Sci Adv. 2024 Nov 8; 10(45):eadp1764. doi: 10.1126/sciadv.adp1764 39504378 PMC11540037

[114] Martinez D, Burgués J, Marco S. Fast Measurements with MOX Sensors: A Least-Squares Approach to Blind Deconvolution. Sensors. 2019 Jan;19(18):4029. doi: 10.3390/s19184029 31540524 PMC6766816

[115] Monroy JG, González-Jiménez J, Blanco JL. Overcoming the Slow Recovery of MOX Gas Sensors through a System Modeling Approach. Sensors. 2012 Oct;12(10):13664–80. doi: 10.3390/s121013664 23202015 PMC3545586

[116] Schmuker M, Bahr V, Huerta R. Exploiting plume structure to decode gas source distance using metal-oxide gas sensors. Sens Actuators B. 2016 Nov;235:636–46. doi: 10.1016/j.snb.2016.05.098

[117] Stetter JR, Jurs PC, Rose SL. Detection of hazardous gases and vapors: pattern recognition analysis of data from an electrochemical sensor array. Anal Chem. 1986 Apr 1;58(4):860–6. doi: 10.1021/ac00295a047

[118] Wilson AD, Baietto M. Applications and Advances in Electronic-Nose Technologies. Sensors (Basel). 2009 Jun 29;9(7):5099–148. doi: 10.3390/s90705099 22346690 PMC3274163

[119] Baker KL, Dickinson M, Findley TM, Gire DH, Louis M, Suver MP, et al. Algorithms for Olfactory Search across Species. J Neurosci. 2018;38(44):9383–9. doi: 10.1523/JNEUROSCI.1668-18.2018 30381430 PMC6209839

[120] xing Chen X, Huang J. Odor source localization algorithms on mobile robots: A review and future outlook. Rob Auton Syst. 2019 Feb 1;112:123–36. doi: 10.1016/j.robot.2018.11.014

[121] Lilienthal AJ, Loutfi A, Duckett T. Airborne Chemical Sensing with Mobile Robots. Sensors. 2006 Nov 20;6(11):1616–78. doi: 10.3390/s6111616

[122] Song HW, Moon D, Won Y, Cha YK, Yoo J, Park TH, et al. A pattern recognition artificial olfactory system based on human olfactory receptors and organic synaptic devices. Sci Adv. 2024 May 23;10(21):eadl2882. doi: 10.1126/sciadv.adl2882 38781346 PMC11114221

[123] Singh SH, van Breugel F, Rao RPN, Brunton BW. Emergent behaviour and neural dynamics in artificial agents tracking odour plumes. Nat Mach Intell. 2023 Jan;5(1):58–70. doi: 10.1038/s42256-022-00599-w 37886259 PMC10601839

[124] Shor E, Herrero-Vidal P, Dewan A, Uguz I, Curto VF, Malliaras GG, et al. Sensitive and robust chemical detection using an olfactory brain-computer interface. Biosens Bioelectron. 2022 Jan;195:113664. doi: 10.1016/j.bios.2021.113664 34624799

[125] Zhang Y, Rózsa M, Liang Y, Bushey D, Wei Z, Zheng J, et al. Fast and sensitive GCaMP calcium indicators for imaging neural populations. Nature. 2023 Mar 15;1–8. doi: 10.1038/s41586-023-05828-9 36922596 PMC10060165

